# Spinal Cord Stimulation for Painful Diabetic Neuropathy: A Systematic Review and Meta‐Analysis of High‐ and Low‐Frequency Modalities

**DOI:** 10.1155/prm/9347376

**Published:** 2026-05-25

**Authors:** Chengjiang Liu, Zidi Xu, Yifu Liang, Yujia Luo, Siyao Zheng, Zhikang Ye, Zhiying Feng

**Affiliations:** ^1^ Department of Pain Medicine, The First Affiliated Hospital, Zhejiang University School of Medicine, Hangzhou, Zhejiang, China, zju.edu.cn; ^2^ Michael G. DeGroote National Pain Centre, McMaster University, Hamilton, Ontario, Canada, mcmaster.ca

**Keywords:** diabetic neuralgia, diabetic neuropathy, nerve pain, neuropathic pain, painful diabetic neuropathy, spinal cord stimulation

## Abstract

**Objective:**

To systematically evaluate the efficacy and safety of high‐frequency (HF‐SCS) and low‐frequency (LF‐SCS) spinal cord stimulation for painful diabetic neuropathy (PDN) and to qualitatively explore potential differences between HF‐SCS and LF‐SCS based on indirect comparisons.

**Methods:**

A systematic review and meta‐analysis were conducted following PRISMA guidelines. We searched PubMed, Scopus, Embase, Web of Science, and the Cochrane Library for studies published between 2015 and 2024. Inclusion criteria included studies involving adult patients with PDN receiving either HF‐SCS or LF‐SCS. Eligible studies reported outcomes such as pain relief VAS, EQ‐5D, and adverse events. Data were analyzed using a random‐effects model. Pain intensity outcomes were converted to a 0–10 scale and pooled as mean differences (MDs). The EQ‐5D index was pooled as standardized mean differences (SMDs). Sensitivity and subgroup analyses were performed to assess heterogeneity.

**Results:**

In total, 9 studies, including 416 participants, were eligible. Moderate certainty evidence suggests that both HF‐SCS and LF‐SCS significantly reduce pain in patients with PDN. In RCTs, SCS reduced pain at 6 months compared with best medical therapy (overall MD on a 0–10 scale: −3.95, 95% CI −5.57 to −2.33). In subgroup analyses, the HF‐SCS trial showed an MD of −5.20 (95% CI −5.79 to −4.61), and LF‐SCS trials showed a pooled MD of −3.22 (95% CI −4.37 to −2.07). Both treatments were associated with low adverse event rates.

**Conclusion:**

Both HF‐SCS and LF‐SCS were associated with substantial reductions in pain intensity in patients with PDN compared with best medical therapy or baseline, although the overall certainty of evidence was at most moderate. Because no head‐to‐head trials directly comparing HF‐SCS and LF‐SCS in PDN were available, comparative effectiveness between modalities remains uncertain; however, these cross‐study differences should be interpreted cautiously because the modalities were not compared head‐to‐head. Improvements in EQ‐5D were modest and did not consistently exceed the prespecified minimal important difference, and both modalities showed low rates of mainly device‐related complications with few serious adverse events reported.

## 1. Introduction

Diabetic neuropathy is one of the most common complications of diabetes, affecting more than half of diabetic patients globally [1]. In 2021, an estimated 537 million adults were living with diabetes worldwide (10.5% prevalence), projected to reach 783 million by 2045 [2]. Painful diabetic neuropathy (PDN) typically manifests as significant sensory, motor, and autonomic dysfunction [3], accompanied by limb pain, numbness, and burning sensations [4]. These symptoms severely impair the quality of life, adversely affect mental health [5], and impose burdens on patients and their families [6].

Traditionally, PDN treatment primarily relies on medications [7]. Early clinical use involved gabapentinoids, serotonin‐norepinephrine reuptake inhibitors, and tricyclic antidepressants [8, 9]. However, some patients develop drug tolerance [10], making pain relief difficult to achieve [11]. Although the U.S. Food and Drug Administration (FDA) recently approved several medications specifically for PDN [12], their overall efficacy remains unsatisfactory [13, 14].

Spinal cord stimulation (SCS) is a neuromodulation technique that intervenes in pain transmission pathways by delivering electrical impulses [15]. It has been used for chronic pain treatment since the late 1960s [16]. Despite multiple proposed mechanisms, the precise mode of action of SCS remains unclear. In recent years, however, SCS has demonstrated significant potential for PDN patients refractory to conventional treatments [17]. Traditional low‐frequency spinal cord stimulation (LF‐SCS, 40–60 Hz) produces analgesia by stimulating the dorsal column of the spinal cord, eliciting a paresthesia sensation in the affected region. In contrast, high‐frequency spinal cord stimulation (HF‐SCS, 10 kHz) achieves analgesic effects without inducing paresthesia [18]. Different SCS modalities have effectively improved symptoms in various chronic pain conditions [15, 19, 20].

Although several studies have compared the efficacy of HF‐SCS and LF‐SCS in treating chronic pain conditions [21–23], direct head‐to‐head randomized controlled trials (RCTs) in PDN are still limited. Duarte et al. reported clinically meaningful reductions in pain intensity at 6 months with SCS compared to medical therapy [24]. In 2022, a network meta‐analysis (NMA) by Duarte et al. further demonstrated that both LF‐SCS and HF‐SCS provide superior pain control versus conventional medical management (CMM) [25]. However, none of these reviews incorporated the most recent evidence from HF‐SCS trials or long‐term follow‐up beyond 6 months. Previous meta‐analyses have explored this issue in recent years, but they were limited by the small number of RCTs available [25]. The present study aims to build upon the existing literature by including newly available 12‐month follow‐up data and observational cohort evidence, thereby providing a more comprehensive assessment of SCS outcomes in PDN.

## 2. Materials and Methods

### 2.1. Data Sources and Search Strategy

This study was conducted according to the Preferred Reporting Items for Systematic Reviews and Meta‐Analyses (PRISMA) guidelines [26]. The review protocol was registered with PROSPERO (Registration Number: CRD420251004444). We systematically searched PubMed, Scopus, Embase, Web of Science, and the Cochrane Library databases. The literature search was performed on February 1, 2025, covering studies published from January 1, 2015, to December 31, 2024. Detailed search strategies are provided in Supporting file 1, Table S1.

### 2.2. Inclusion and Exclusion Criteria

Included studies involved adult patients (≥ 18 years) diagnosed with PDN who received either LF‐SCS or HF‐SCS. Studies were eligible if they reported outcomes relevant to pain relief and quality of life, such as Visual Analog Scale (VAS), Numeric Rating Scale (NRS), or EuroQol 5‐Dimensions (EQ‐5D), as well as adverse events. The included studies were either RCT or cohort studies. The minimum required follow‐up duration for pain outcomes was 6 or 12 months. Studies were excluded if they provided insufficient or incomplete data, did not report the outcomes of interest clearly, or included an extremely small sample size (fewer than three patients). In the included RCTs, the control groups were variably labeled as best medical treatment (BMT) or CMM. Due to the absence of direct head‐to‐head RCTs directly comparing HF‐SCS and LF‐SCS for PDN, this meta‐analysis first separately evaluated the efficacy and safety of each stimulation modality in comparison with BMT or baseline conditions. Subsequently, we qualitatively explored patterns across HF‐SCS and LF‐SCS trials by comparing their respective pooled estimates. We did not conduct a formal NMA due to the limited number of available studies and insufficient direct or indirect comparative data to satisfy the assumptions required for robust NMA modeling.

## 3. Literature Screening and Data Extraction

Two independent reviewers (Liu and Xu) screened all retrieved articles and extracted data, cross‐verifying the results. Any discrepancies were resolved by discussion or a third reviewer (Feng). Data extracted included the first author’s name, publication year, study design, sample size, randomization and blinding methods (for RCTs), intervention details (LF‐SCS or HF‐SCS), follow‐up duration, and primary outcome measures. Data were cross‐checked between the two reviewers to ensure accuracy and consistency.

### 3.1. Risk‐of‐Bias Assessment

**FIGURE 1:**
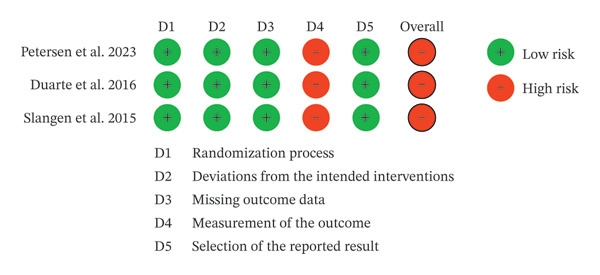
Analysis of risk of bias using the Cochrane RoB‐2 tool.

For RCTs, two reviewers independently assessed risk of bias using the Cochrane Risk of Bias 2 tool (RoB 2) at the outcome level for the prespecified critical outcomes (pain intensity at 6 months; EQ‐5D index at 6 months) [27] (Figure 1). RoB 2 evaluates five domains: (D1) bias arising from the randomization process; (D2) bias due to deviations from intended interventions (including appropriateness of intention‐to‐treat analyses and potential impact of non‐adherence/crossover); (D3) bias due to missing outcome data; (D4) bias in measurement of the outcome (open‐label device trials with patient‐reported outcomes); and (D5) bias in selection of the reported result. Each domain was judged as “low risk,” “some concerns,” or “high risk,” and an overall RoB judgment was derived using the RoB 2 algorithm. Disagreements were resolved by discussion with a third reviewer. Detailed assessments and outcomes are presented in Supporting file 1, Table S2.

The Newcastle–Ottawa Scale (NOS) was utilized to assess the quality of cohort studies [28] (Table 1). NOS evaluates study quality based on three dimensions: participant selection, comparability of study groups, and outcome measurement, comprising a total of nine items with a maximum score of nine points. Studies were categorized as high quality (scores of 7–9), moderate quality (4–6), or low quality (≤ 3).

**TABLE 1:** Cohort study quality rating.

Author	Selection	Comparability	Exposure/outcome	Total
Cyrek et al. (2024)	2	2	3	7
Chen et al. (2023)	1	0	1	2
Kissoon et al. (2023)	1	0	2	3
Yan et al. (2021)	1	0	1	2
Galan et al. (2020)	1	0	2	3
van Beek et al. (2018)	2	0	2	4

*Note:* Quality of cohort studies was determined using the Newcastle–Ottawa Scale, which evaluates three categories: selection (maximum 4), comparability (maximum 2), and outcome (maximum 3).

### 3.2. Quality Assessment

We assessed certainty of evidence using the Grading of Recommendations Assessment, Development, and Evaluation (GRADE) approach for critical outcomes [29, 30]. Evidence from RCTs started as high certainty, and observational cohort evidence started as low certainty. We considered downgrading for the five GRADE domains: (1) risk of bias (RoB 2 for RCTs; NOS for cohorts); (2) inconsistency (unexplained heterogeneity in magnitude or direction of effects; *I* ^2^ used as supportive information); (3) indirectness (differences in populations, interventions, comparators, outcomes, or measurement instruments); (4) imprecision (width of confidence intervals and whether the CI crossed a prespecified clinically important threshold); and (5) publication bias (not formally assessed when < 10 studies were available). To support clinical interpretation, we prespecified minimal important differences (MID): A reduction of ≥ 2 points on a 0–10 pain scale and an increase of ≥ 0.10 on the EQ‐5D utility index were considered clinically meaningful [31].

### 3.3. Data Analysis

Meta‐analysis was conducted using RevMan 5.4 and R 4.2.1 software. Pain intensity outcomes (VAS/NRS) were converted to a 0–10 scale and pooled as mean differences (MDs) using a random‐effects model. EQ‐5D index outcomes were pooled as standardized mean differences (SMDs). Heterogeneity among studies was assessed using the I^2^ statistic. Subgroup and sensitivity analyses were performed when significant heterogeneity was observed (*I* ^2^ ≥ 50%). This study performed a leave‐one‐out analysis by sequentially excluding individual studies to assess their impact on heterogeneity and the pooled effect size. Given the limited number of studies, if exclusion of one study resulted in insufficient subgroup data, the sensitivity analysis was not performed for that subgroup, and this limitation was explicitly acknowledged.

Primary outcomes analyzed included pain relief (changes in pain scores at 6 months in RCTs and at 6 or 12 months in cohort studies), quality‐of‐life improvement (EQ‐5D index at 6 months in RCTs), and adverse event incidence (comparing complications between HF‐SCS and LF‐SCS at 6 months). All pain scores were converted to a 0–10 scale using linear proportional conversion. When conversion was required, both the mean and standard deviation were rescaled by the same factor (SD_new = SD_old × 10/scale_max) before pooling MDs. Because pain outcomes were pooled as MD on a 0–10 scale, the pooled effect estimates are directly interpretable without additional back‐transformation [32]. Although van Beek et al. met inclusion criteria, it did not provide extractable 6‐ or 12‐month VAS/NRS data in the format required for our prespecified forest plots [33]. As a result, it was not included in the forest plot comparison.

## 4. Results

### 4.1. Literature Search

**FIGURE 2:**
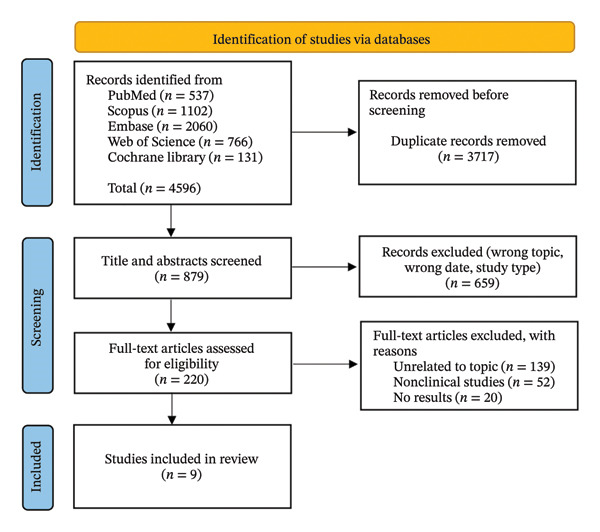
PRISMA flow diagram of study selection.

The initial search yielded 4596 references (Figure 2). After screening, 9 articles (3 RCTs and 6 cohort studies) met the inclusion criteria [19, 33–40]. Characteristics of the included studies are summarized in Table 2. Among these, four studies evaluated HF‐SCS (1 RCT), and five evaluated LF‐SCS (2 RCTs). A total of 416 patients were included, of whom 305 underwent SCS implantation (HF‐SCS: *n* = 179; LF‐SCS: *n* = 126), with follow‐up durations ranging from 1 month to 5 years.

**TABLE 2:** Characteristics of included studies.

Study	Study design	*n*	Follow‐up (months)	Adverse events	Limitation	Intervention	Outcomes measures
Cyrek et al. [34]	Retrospective cohort study	13	12	No report	Small sample size, single‐center retrospective study	Low‐frequency	Visual Analog Scale (VAS) was used to measure perceived pain
Chen et al. [19]	Prospective cohort study	8	12	No report	Small sample size. Non‐blind method	High‐frequency (10 kHz)	Numeric rating scale for pain, Neuropathic Pain Scale (NPS)
Kissoon et al. [35]	Prospective cohort study	10	12	No report	Small sample size. Nonrandomized	High‐frequency (10 kHz)	Assessments of pain intensity were made with the VAS
Petersen et al. [36]	RCT	216	24	The infection rate was 5.2% (8/154), 3.2% (5/154) required device removal	Nonblind design, crossover design	High‐frequency (10 kHz)	The percentage of participants with 50% pain relief on VAS
Yan et al. [37]	Retrospective cohort study	16	12	Electrode displacement: 6 cases (42.8%). Delayed wound healing: 2 cases (14.2%)	This is a retrospective study with a small sample size	Low‐frequency	Pain intensity using a VAS
Galan et al. [38]	Prospective cohort study	9	12	5 cases (lower limb pain, seroma, implant site dehiscence, liver failure)	Small sample size, lack of control group	High‐frequency (10 kHz)	Pain intensity using a VAS
van Beek et al. [33]	Prospective cohort study	48	60	Infection 2 cases, lead adjustment 9 cases, subcutaneous battery discomfort 10 cases	The sample size was small, there was no control group	Low‐frequency	NRS score for pain during the day and night was the primary outcome
Duarte et al. [39]	RCT	60	6	No report	Lack of blind design, small sample size	Low‐frequency	Pain intensity using a VAS
Slangen et al. [40]	RCT	36	24	One patient died of subdural hematoma, one patient had infection	Small sample size, no blinding	Low‐frequency	Pain intensity using a NRS

### 4.2. Risk of Bias in Included RCTs

D1 (randomization process) was judged as low risk because randomization and allocation concealment were reported and baseline imbalances did not suggest problems with randomization. D2 (deviations from intended interventions) was judged as low risk because analyses were conducted using intention‐to‐treat principles and no clinically important deviations from assigned interventions were reported during the 6‐month outcome window; the crossover feature in the HF‐SCS trial occurred after the primary 6‐month comparison and therefore did not affect the 6‐month effect estimate. D3 (missing outcome data) was judged as low risk because outcome data were available for nearly all randomized participants and missingness was unlikely to depend on the true outcome. D5 (selection of the reported result) was judged as low risk because prespecified outcomes and time points were reported without clear evidence of selective reporting. However, D4 (measurement of the outcome) was judged as high risk for the key outcomes because blinding was not feasible in trials comparing an implanted neuromodulation device with medical management, and both pain intensity (VAS/NRS) and EQ‐5D are patient‐reported outcomes that may be influenced by participants’ awareness of treatment assignment. Consequently, the overall RoB 2 judgment was high for all included RCTs. Figure 1 and Supporting Table S2 summarize RoB 2 assessments for the three included RCTs.

### 4.3. Pain Intensity

Among three RCTs, both HF‐SCS and LF‐SCS were associated with clinically meaningful pain reductions compared with best medical therapy (Figure 3(a)). The single HF‐SCS trial (Petersen 2023) reported a large reduction at 6 months (MD on a 0–10 scale), and the two LF‐SCS trials [39, 40] also showed significant reductions. Because HF‐SCS and LF‐SCS were not compared head‐to‐head and differed across trials in design and populations, we did not perform a formal between‐modality comparison; differences in point estimates should be interpreted as hypothesis‐generating. Due to the presence of only one HF‐SCS study [36], sensitivity analysis could not be performed.

FIGURE 3Forest plots comparing clinical outcomes of SCS across study designs. (a) VAS scores in RCTs comparing SCS with BMT; (b) EQ‐5D scores in RCTs comparing SCS with BMT; (c) 6‐month VAS score changes from baseline in cohort studies evaluating SCS efficacy; (d) 12‐month VAS score changes from baseline in cohort studies evaluating SCS efficacy.

**Figure:**
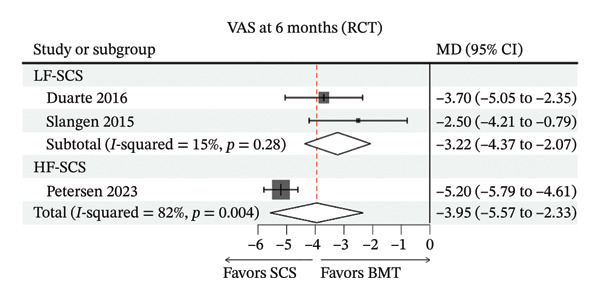
(a)

**Figure:**
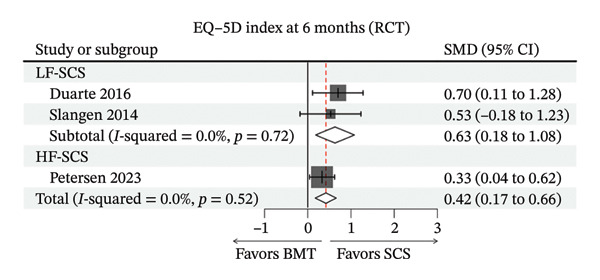
(b)

**Figure:**
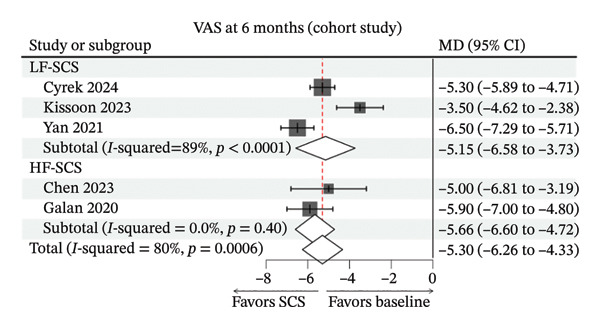
(c)

**Figure:**
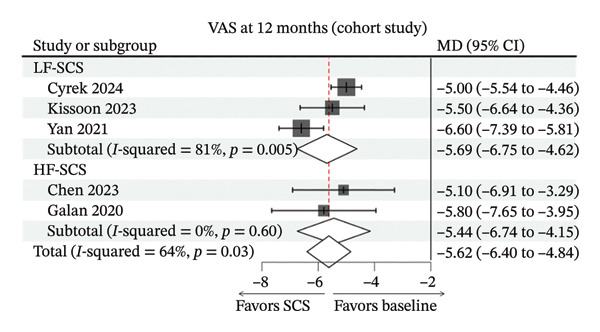
(d)

In cohort studies, SCS was associated with substantial pain reductions from baseline. At 6 months, the pooled change was MD −5.30 (95% CI −6.26 to −4.33; *I* ^2^ = 80%). In subgroup analyses, LF‐SCS showed MD −5.15 (95% CI −6.58 to −3.73; *I* ^2^ = 89%), and HF‐SCS showed MD −5.66 (95% CI −6.60 to −4.72; *I* ^2^ = 0%) (Figure 3(c)). At 12 months, the pooled change was MD −5.62 (95% CI −6.40 to −4.84; *I* ^2^ = 64%). LF‐SCS showed MD −5.69 (95% CI −6.75 to −4.62; I^2^ = 81%), and HF‐SCS showed MD −5.44 (95% CI −6.74 to −4.15; *I* ^2^ = 0%) (Figure 3(d)). Because these were nonrandomized cohorts without head‐to‐head comparisons and differed across studies, frequency‐stratified estimates should not be interpreted as evidence of modality superiority. Detailed sensitivity analysis results can be found in Supporting file 1, Figure S1.

### 4.4. Quality of Life

Regarding EQ‐5D scores at 6 months (Figure 3(b)), the LF‐SCS group had an SMD of 0.63 (95% CI: 0.18–1.08), no heterogeneity was observed in this subgroup (*I* ^2^ = 0%, *p* = 0.72). The HF‐SCS group showed an SMD of 0.33 (95% CI: 0.04–0.62). The pooled result indicated significant quality‐of‐life improvement following SCS treatment (SMD = 0.42, 95% CI: 0.17–0.66). No heterogeneity was observed (*I* ^2^ = 0%, *p* = 0.52).

### 4.5. Adverse Events

Due to incomplete reporting of adverse events in included studies, a quantitative meta‐analysis was not feasible, and a descriptive approach was adopted. Common adverse events included infection, electrode migration or fracture, and pain at the implantation site. Adverse event reporting was incomplete and heterogeneous across studies. Therefore, we did not pool adverse events quantitatively. Commonly reported events included infection, wound‐related complications, lead migration/revision, and implant‐site discomfort. Given heterogeneous and incomplete adverse event reporting across studies, we summarized adverse events descriptively by study and modality and did not perform a quantitative between‐modality comparison (Supporting file 1, Table S3). Serious complications were uncommon; however, one LF‐SCS RCT reported a fatal subdural hematoma following dural puncture. Given heterogeneous and incomplete adverse‐event reporting across studies, we present adverse events descriptively in Supporting file 1 (Table S3) without quantitative between‐modality comparisons. Limited data suggested minimal differences in surgical safety and device‐related complications between HF‐SCS and LF‐SCS, indicating comparable safety profiles.

### 4.6. Evidence Quality Assessment (GRADE)

Using GRADE, certainty for RCT evidence started at high. For pain intensity at 6 months, we downgraded one level for risk of bias because all RCTs were open‐label device trials and the primary outcome was patient‐reported (RoB 2 domain D4 judged high). We did not downgrade for imprecision because confidence intervals indicated a clinically meaningful reduction exceeding the prespecified MID (≥ 2 points on a 0–10 scale). We did not downgrade for publication bias due to the small number of trials and inability to meaningfully assess funnel plot asymmetry. Although heterogeneity was substantial when pooling all RCTs together, it was largely attributable to prespecified frequency subgroups (HF vs. LF) and differences in trial designs; therefore, we did not further downgrade for inconsistency. Overall certainty for the 6‐month pain outcome was moderate.

For the EQ‐5D index at 6 months, we downgraded one level for risk of bias (open‐label trials with patient‐reported HRQoL) and one level for imprecision relative to the MID, because the pooled confidence interval included improvements smaller than the prespecified clinically meaningful threshold (MID = 0.10). Overall certainty for EQ‐5D was therefore low.

For adverse events, certainty was downgraded for risk of bias (incomplete and heterogeneous AE reporting across studies) and serious imprecision (rare events and wide confidence intervals), resulting in low/very‐low certainty depending on study design (Tables 3 and 4).

**TABLE 3:** GRADE summary of findings for BMT versus SCS in patients from RCTs.

Outcomes	Absolute effect estimates			Certainty of evidence	Plain language summary
	Relative effects (95% CI)	Baseline risk of control group	Difference (95% CI)		
6 months VAS	MD −3.95 (−5.57 to −2.33) 270 patients in three trials	7.03	−3.95 (−5.57 to −2.33)	Moderate (downgraded 1 for RoB)	There is probably a big decrease in VAS with SCS
Adverse events	OR8.15 (1.01–66.00) 270 patients in three trials	Not applicable	5% (1%–10%)	Low (downgraded for RoB and imprecision)	There is possibly a small increase in complications with SCS
EQ‐5D index	SMD 0.42 (0.17–0.66) 270 patients in three trials	0.65	0.097 (0.085–0.108)	Low (downgraded 1 for RoB, 1 for imprecision relative to MID)	The EQ‐5D index of SCS may increase slightly

*Note:* EuroQol 5‐Dimensions (EQ‐5D). Downgraded 1 level for risk of bias: trials were open‐label and outcomes were patient‐reported; RoB 2 domain D4 was judged high. Downgraded 1 level for imprecision (EQ‐5D): the 95% CI included improvements smaller than the prespecified MID (0.10). Downgraded for risk of bias and serious imprecision (adverse events): AE reporting was incomplete/heterogeneous, and events were rare with wide CIs.

Abbreviations: SCS, spinal cord stimulation; VAS, Visual Analog Scale.

**TABLE 4:** GRADE summary of findings for baseline versus SCS in patients from cohort studies.

Outcomes	Absolute effect estimates			Certainty of evidence	Plain language summary
	Relative effects (95% CI)	Baseline risk of control group	Difference (95% CI)		
6 months VAS	MD −5.30 (−6.26 to −4.33) 103 patients in five studies	7.03	−5.46 (−7.47 to −3.46)	Low (observational evidence; uncontrolled pre–post design)	There is probably a big decrease in VAS with SCS
12 month VAS	MD −5.62 (−6.40 to −4.84) 101 patients in five studies	7.03	−5.49 (−7.26 to −3.74)	Low (observational evidence; uncontrolled pre–post design)	There is probably a big decrease in VAS with SCS
Adverse events	OR23.15 (2.73–196.13) 103 patients in five studies	Not applicable	22% (10%–34%)	Very low (serious imprecision and risk of bias)	There is possibly a small increase in complications with SCS

Abbreviations: SCS, spinal cord stimulation; VAS, Visual Analog Scale.

For cohort studies, initial certainty was low due to selection bias, small sample sizes, and the absence of controls. Despite the significant pain relief observed at 6 and 12 months (pooled MD −5.30 and −5.62 on a 0–10 scale, respectively), certainty remained low due to the observational design and risk of bias (Table 4).

## 5. Discussion

This meta‐analysis showed that both LF‐SCS and HF‐SCS can significantly relieve pain in patients with PDN while slightly improving quality of life. Although EQ‐5D scores improved statistically after SCS, the pooled mean change (∼0.10 from a baseline of 0.65) was at the boundary of, and in some analyses below, the prespecified MID. Thus, the clinical relevance of quality‐of‐life gains remains uncertain. Both treatments also had a favorable safety profile, with low adverse event rates, though LF‐SCS was associated with slightly more issues such as electrode migration and wound healing complications. GRADE assessment indicated that the evidence for pain reduction was of moderate certainty for RCTs and low for cohort studies. Our review differs from previous studies in that we did not attempt a formal indirect comparison; instead, we presented the two models side by side with their respective controls. This approach avoids potential biases in cross‐trial comparisons and reflects the reality that no head‐to‐head RCT evidence exists yet. Nonetheless, our narrative comparison of the separate results is broadly consistent with the NMA: When the two modalities are considered side by side, both HF‐SCS and LF‐SCS show large, clinically relevant pain reductions versus medical therapy. Across separate trials, both HF‐SCS and LF‐SCS showed large, clinically meaningful pain reductions versus their respective comparators; however, the relative effectiveness between modalities remains uncertain because no head‐to‐head randomized evidence is available. By incorporating 12‐month follow‐up and an observational study, our analysis also adds new insight into the durability of SCS benefits, an area not covered in prior reviews. Thus, this study reinforces the consensus that SCS is effective for PDN and highlights the ongoing need for further research to refine its use.

### 5.1. Pain Relief and Efficacy Comparison

Both HF‐SCS and LF‐SCS demonstrated substantial pain relief in patients with PDN across randomized and observational evidence. In the available RCTs, the HF‐SCS evidence is currently driven by a single trial, while LF‐SCS evidence comes from two separate trials; therefore, any apparent differences in effect size across modalities reflect between‐trial rather than within‐trial comparisons. Trial‐level differences may confound cross‐study contrasts. Accordingly, we interpret the pooled estimates for HF‐SCS and LF‐SCS as evidence that each modality is effective compared with its respective comparator, while the comparative effectiveness between modalities remains uncertain and should be addressed by future head‐to‐head trials. LF‐SCS analgesia predominantly follows Melzack and Wall’s gate control theory (1965), suggesting electrical stimulation activates A‐beta sensory fibers, thus suppressing pain transmission through inhibitory interneurons affecting A‐gamma and C‐fibers [41]. Clinically, LF‐SCS employs low frequencies (40–60 Hz), amplitudes of 2–8 mA, and pulse widths around 200–500 µs [42], producing paresthesia covering painful areas. In contrast, HF‐SCS produces analgesia without paresthesia, indicating an analgesic mechanism not reliant on A‐beta fiber activation [43]. Research suggests HF‐SCS activates inhibitory interneurons without directly stimulating dorsal horn sensory fibers responsible for paresthesia [44]. Additional potential analgesic mechanisms involve neurotransmitters such as gamma‐aminobutyric acid (GABA), glial cells, and anti‐inflammatory cytokines [45, 46].

An important message from our review is that LF‐SCS and HF‐SCS should be viewed as complementary modalities in the therapeutic arsenal for PDN, rather than opposing options where one must be chosen over the other. Traditional tonic SCS has a decades‐long track record, with well‐established programming paradigms and many patients worldwide treated, whereas HF‐SCS is a newer innovation offering specific advantages such as paresthesia‐free stimulation. In practice, the choice between HF‐SCS and LF‐SCS may depend on individual patient factors and preferences. Some patients find paresthesias tolerable or even reassuring, and they may do well with conventional LF‐SCS. Others cannot tolerate the tingling sensations or do not achieve sufficient relief with tonic SCS—these individuals could benefit from the HF‐SCS approach, which produces no paresthesia and has shown excellent pain relief outcomes. Additionally, device availability and clinician experience can influence modality selection: Certain centers may have more expertise with one system or the other. Rather than viewing HF‐SCS as a wholesale replacement for LF‐SCS, it is better seen as an expansion of options. Our data suggest that both modalities are effective compared to medical therapy, so having both available allows a tailored, patient‐centric approach—a cornerstone of modern pain management. It is conceivable that future treatment algorithms will integrate both HF‐SCS and LF‐SCS, using criteria such as pain distribution, comorbidities, or even biomarker information to decide which stimulation pattern to initiate for a given patient. At this stage, we encourage clinicians to become familiar with both technologies, as each has a role to play in managing refractory PDN.

### 5.2. Adverse Events

In studies of HF‐SCS, Petersen et al. reported 5.2% (8 patients) infection, with 3.2% (5 patients) requiring explantation and 1.9% (3 patients) needing re‐implantation or adjustment of the implanted pulse generator or electrodes [36]. Galan et al. observed mild pain at the implant site in 1 patient, hematoma at the implant site in 1 patient, and wound dehiscence in 1 patient [38]. For LF‐SCS, Yan et al. found 42.8% (6 patients) had mild electrode migration, corrected by stimulation adjustments, and 14.2% (2 patients) had delayed wound healing [37]. Slangen et al. reported a fatal subdural hematoma in 1 patient following dural puncture and an infection in 1 patient requiring explantation [40]. Cyrek et al. noted 30.77% (4 patients) needed device replacement due to battery depletion, with no issues related to device failure [34]. van Beek et al. reported 4.2% (2 patients) infections and 18.8% (9 patients) lead adjustments [33]. Both HF‐SCS and LF‐SCS had low adverse event rates, but LF‐SCS was associated with more issues like electrode displacement and wound healing. Across HF‐SCS and LF‐SCS studies, serious neurological complications were rare, and most adverse events were manageable device‐ or procedure‐related problems such as infection or lead migration, supporting an overall favorable short‐to mid‐term safety profile for both modalities.

### 5.3. Future Research Directions

Given the relatively recent introduction of HF‐SCS, its long‐term effectiveness and safety remain under investigation. Important questions include the development of patient tolerance or potential device replacement due to long‐term use. Extended follow‐up studies may further clarify whether SCS impacts PDN progression, including neuropathy severity or glycemic control. Future research should explore novel technologies beyond frequency optimization [47]. Closed‐loop SCS systems, capable of real‐time monitoring and automatic stimulation adjustments, have demonstrated sustained analgesic effectiveness in other chronic pain conditions and should be evaluated in PDN [48]. Additionally, emerging stimulation patterns such as burst waveforms offer promising therapeutic options for refractory PDN [49].

### 5.4. Study Limitations

We acknowledge several limitations in our review. First, the number of RCTs available for inclusion was small, reflecting the still‐emerging evidence base for SCS in PDN. This limited our ability to conduct subgroup analyses or meta‐regressions, and results should be interpreted with caution given the potential for publication bias. Several pooled analyses, particularly those for 6‐month pain outcomes, showed substantial statistical heterogeneity. These high *I* ^2^ values indicate that a large proportion of the between‐study variability is unlikely to be due to chance alone and probably reflects differences in study design, patient selection, and stimulation protocols. Consequently, the pooled MDs should be interpreted as average effects, and the clinical relevance should be considered in relation to the prespecified MID on a 0–10 pain scale. Second, the direct comparability of the HF‐SCS and LF‐SCS trials is limited by differences in study design. We attempted to mitigate this by not pooling them directly, but it still affects any indirect comparisons we discuss. Third, we included an observational cohort study to incorporate 12‐month data; while this enhances the clinical relevance of our review, it introduces a lower level of evidence compared to RCTs and potential biases. We judged that the value of understanding longer‐term outcomes outweighed these concerns, but readers should keep in mind the distinction between the controlled trial evidence and the cohort data. Fourth, our review focused on pain and quality of life outcomes; we did not extensively cover other aspects such as cost‐effectiveness. Finally, although we followed rigorous systematic review methods, the evolving nature of this field means new studies may soon become available that could further inform or change some conclusions. Despite these limitations, we believe our analysis provides a timely and comprehensive synthesis of current evidence to guide clinicians and stakeholders in the management of PDN with neuromodulation.

## 6. Conclusion

Both LF‐SCS and HF‐SCS are effective and relatively safe treatments for PDN that is resistant to CMM. In the available randomized trials, SCS, whether delivered at high or low frequency, reduced pain more than best medical therapy, with clinically meaningful pain reductions observed in both HF‐SCS and LF‐SCS trials. Observational cohort studies also showed large reductions in pain after implantation, with sustained benefits up to 12 months and beyond, although effect estimates varied widely and were at risk of bias. Improvements in EQ‐5D scores were small and did not consistently exceed the prespecified MID, so the clinical significance of quality‐of‐life changes remains uncertain. Adverse events were infrequent and mainly related to infection, lead migration, or wound healing, supporting an overall favorable safety profile for both modalities. Given the small number of studies, heterogeneity in design and populations, and the absence of head‐to‐head comparisons, current evidence does not allow firm conclusions about the relative superiority of HF‐SCS versus LF‐SCS; both should therefore be regarded as viable options, and treatment choice should be individualized.

## Funding

This study was supported by the 2024 Science Research Fund of National Health Commission of China—Major Health & Technology Project of Zhejiang Province (Grant No. WKJ‐ZJ‐2404) and National Natural Science Foundation of China (Grant No. 82271239).

## Ethics Statement

The authors have nothing to report.

## Conflicts of Interest

The authors declare no conflicts of interest.

## Supporting Information

Additional supporting information can be found online in the Supporting Information section.

## Supporting information


**Supporting Information** Supporting file 1 accompanies this manuscript. Supporting Table S1 provides the detailed search strategies for PubMed, Scopus, Embase, Web of Science, and the Cochrane Library. Supporting Table S2 presents the detailed outcome‐level RoB 2 assessments for the included randomized controlled trials. Supporting Table S3 summarizes the study‐level adverse events reported for HF‐SCS and LF‐SCS. Supporting Figure S1 shows the leave‐one‐out sensitivity analyses for pooled pain outcomes.

## Data Availability

All data generated or analyzed during this study are included in this published article/as supporting information files.
