# Supplementary material for: Spinal Cord Stimulation for Painful Diabetic Neuropathy: A Systematic Review and Meta‐Analysis of High‐ and Low‐Frequency Modalities
Source: Pain Res Manag. 2026 May 25;2026:9347376. doi: 10.1155/prm/9347376 (PMC13200277; doi:10.1155/prm/9347376)
Supplement: Supplementary file 1 — Supporting Information Supporting file 1 accompanies this manuscript. Supporting Table S1 provides the detailed search strategies for PubMed, Scopus, Embase, Web of Science, and the Cochrane Library. Supporting Table S2 presents the detailed outcome‐level RoB 2 assessments for the included randomized controlled trials. Supporting Table S3 summarizes the study‐level adverse events reported for HF‐SCS and LF‐SCS. Supporting Figure S1 shows the leave‐one‐out sensitivity analyses for pooled pain outcomes. [file PRM-2026-9347376-s001.docx]

**Supplementary file 1**

This supplementary file contains the following tables and figures

**Table S1** Search strategy

**Table S2** Cochrane Risk of Bias Assessment for randomized controlled trials

**Table S3** Adverse events reported in included studies

**Figure S1** Sensitivity analysis results (A-N)

Table S1 Search strategy

| **Database** | **Search term** |
| --- | --- |
| PubMed | ("Spinal Cord Stimulation" OR "SCS" OR "Neuromodulation") AND ("Diabetic Peripheral Neuropathy" OR "DPN" OR "Diabetes Mellitus Complications" OR "Peripheral Nerve Disease") AND ("2015/01/01"[Date - Publication]: "2024/12/31"[Date - Publication]) |
| Scopus | (TITLE-ABS-KEY ("Spinal Cord Stimulation" OR "SCS" OR "Neuromodulation") AND TITLE-ABS-KEY ("Diabetic Peripheral Neuropathy" OR "DPN" OR "Diabetes Mellitus Complications" OR "Peripheral Nerve Disease")) AND PUBYEAR AFT 2015 AND PUBYEAR BEF 2025 |
| Embase | ('spinal cord stimulation'/exp OR 'SCS'/exp OR 'neuromodulation'/exp) AND ('diabetic peripheral neuropathy'/exp OR 'DPN'/exp OR 'diabetes mellitus complications'/exp OR 'peripheral nerve disease'/exp) AND ('2015'/sd: '2024'/sd) |
| Web of Science | TS=((“Spinal Cord Stimulation" OR "SCS" OR "Neuromodulation") AND ("Diabetic Peripheral Neuropathy" OR "DPN" OR "Diabetes Mellitus Complications" OR "Peripheral Nerve Disease")) AND PY=2015-2024 |
| Cochrane Library | ("Spinal Cord Stimulation" OR "SCS" OR "Neuromodulation") AND ("Diabetic Peripheral Neuropathy" OR "DPN" OR "Diabetes Mellitus Complications" OR "Peripheral Nerve Disease") AND (Publication Date from 2015-01-01 to 2024-12-31) |

Table S2 Cochrane Risk of Bias Assessment for randomized controlled trials


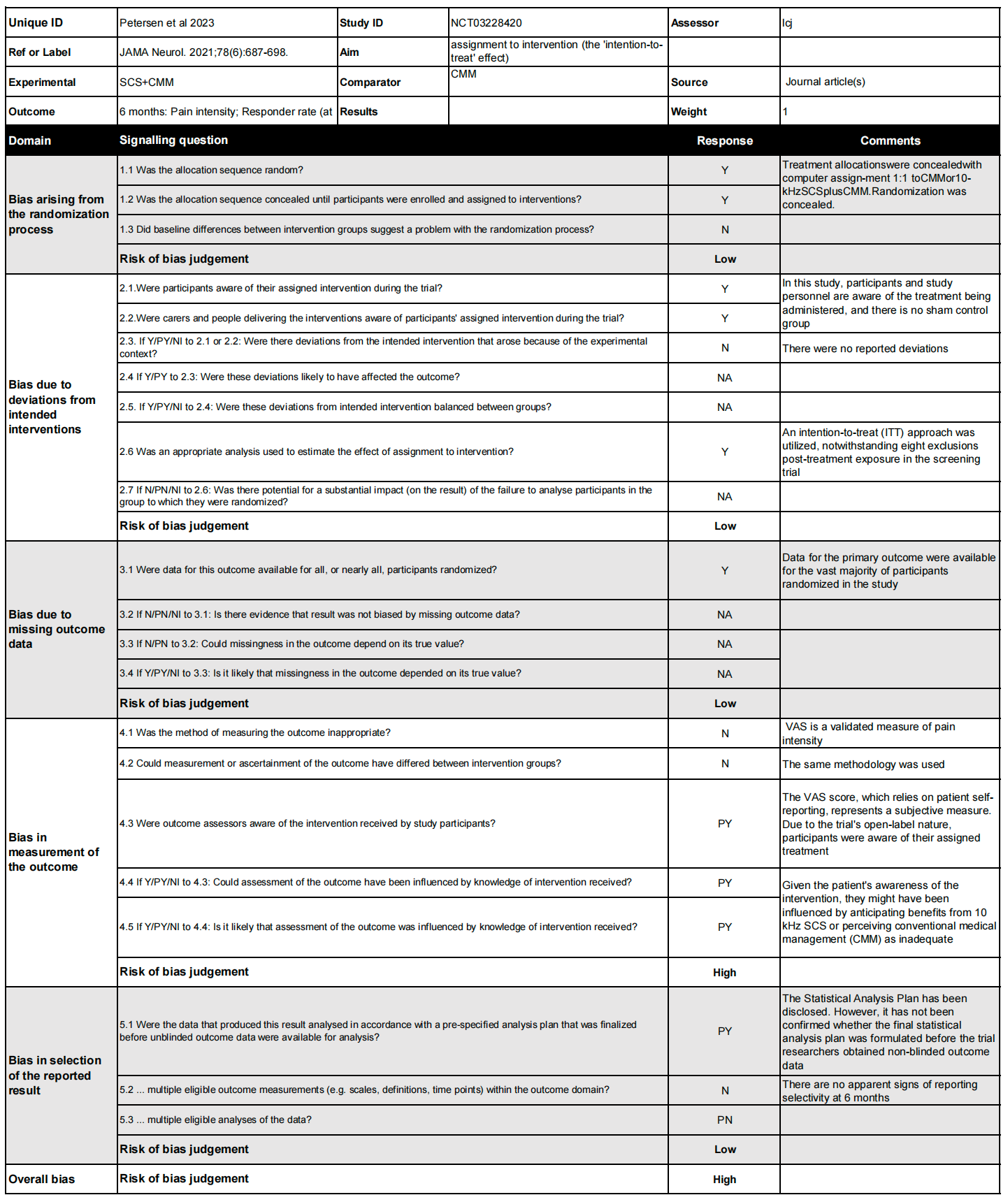


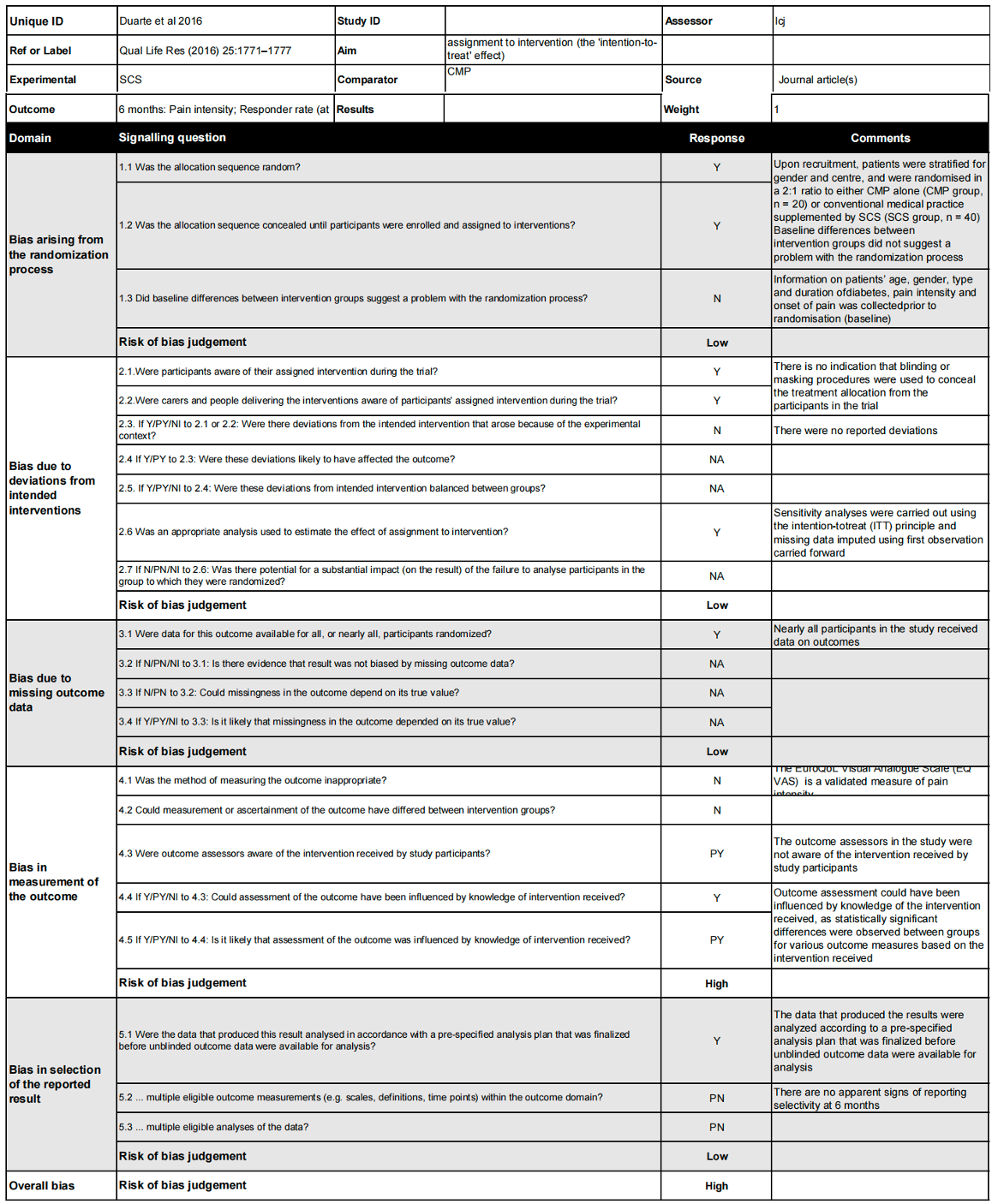


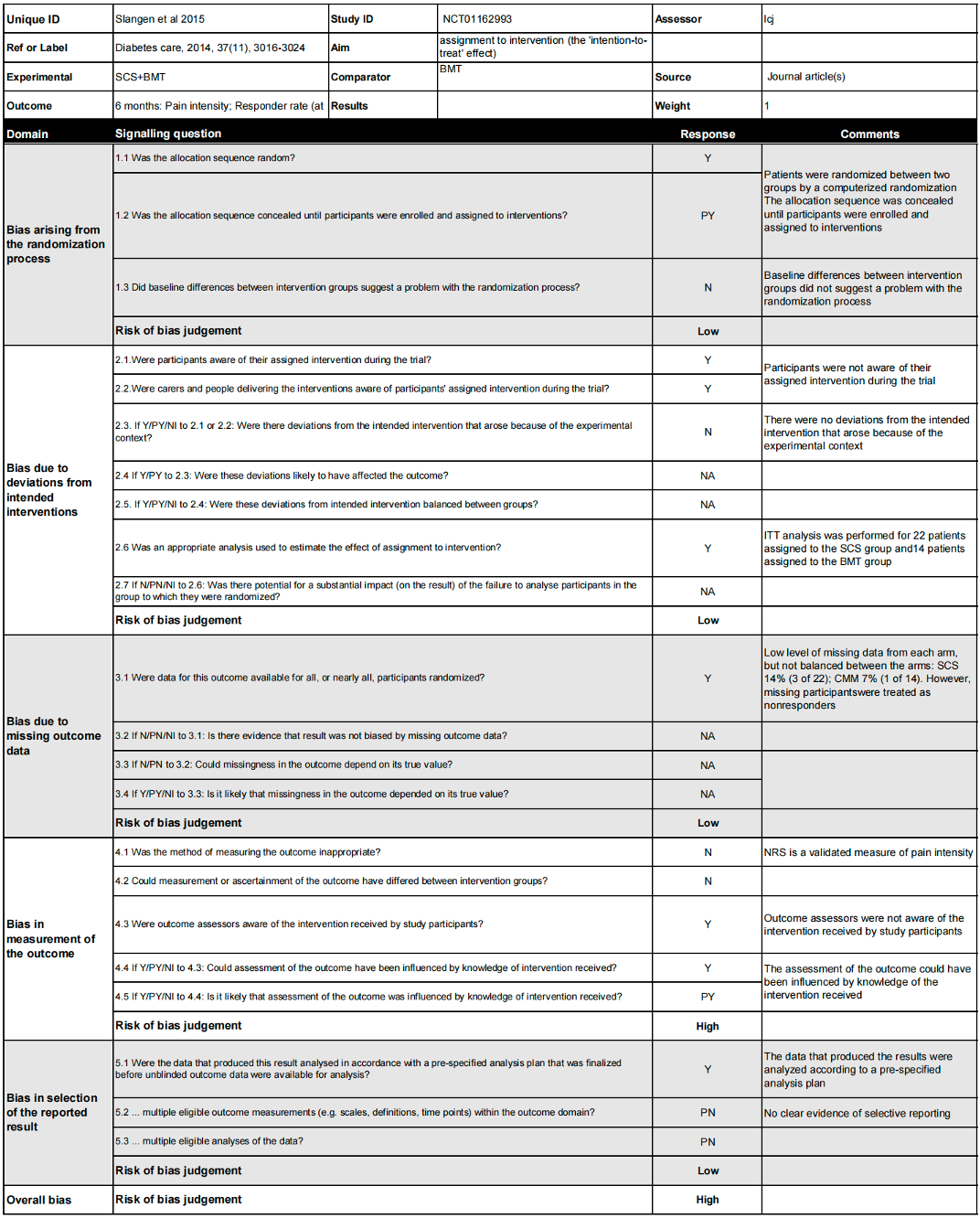


## Table S3 Adverse events reported in included studies

| **Study (year)** | **Design / modality** | **Follow-up** | **Population (n)** | **Reported adverse events (n, % if available)** | **Notes** |
| --- | --- | --- | --- | --- | --- |
| Petersen et al. (2023) | RCT; HF-SCS (10 kHz) + CMM vs CMM | 6 months; 24 months | 6 months: 90 implanted  24 months: 154 implanted | 6 months: 18 AEs in 14 patients; wound-related AEs in 5/90 (6%): infection (3), wound dehiscence (2), impaired healing (1); explant 2/90 (2%). 24 months: infection rate 8/154 (5.2%); infection-related explant 5/154 (3.2%). | CMM arm had no study-related AEs reported in trial protocol. Counts are study-related AEs. |
| Slangen et al. (2015) | RCT; LF-SCS + BMT vs BMT | Up to 24 months | 36 randomized | Death: 1 (subdural hematoma after dural puncture); infection: 1 (required antibiotic treatment and device removal). | AE reporting focused on serious events. |
| Duarte et al. (2016) | RCT; LF-SCS vs CMP | 6 months | 60 randomized | Not reported. | Three patients did not proceed to implantation. |
| van Beek et al. (2018) | Prospective cohort; LF-SCS | 60 months | 48 | Infection: 2; lead adjustment/revision: 9; subcutaneous battery discomfort: 10. | A prospective two-center clinical trial. |
| Cyrek et al. (2024) | Retrospective cohort; LF-SCS | 12 months | 13 | No procedure- or device-related complications reported. | Population is diabetic patients. |
| Yan et al.  (2021) | Retrospective cohort; LF-SCS | 12 months | 16 | Electrode displacement: 6 (42.8%); delayed wound healing: 2 (14.2%). | N/A |
| Galan et al. (2020) | Prospective cohort; HF-SCS (10 kHz) | 12 months | 9 | Five adverse events reported (lower limb pain, seroma, implant-site dehiscence, liver failure). | Small sample; reporting format heterogeneous. |
| Chen et al. (2023) | Prospective cohort; HF-SCS (10 kHz) | 12 months | 8 | Not reported. | N/A |
| Kissoon et al. (2023) | Prospective cohort; HF-SCS (10 kHz) | 12 months | 10 | Not reported. | N/A |

Figure S1 Sensitivity analysis results (A-N)


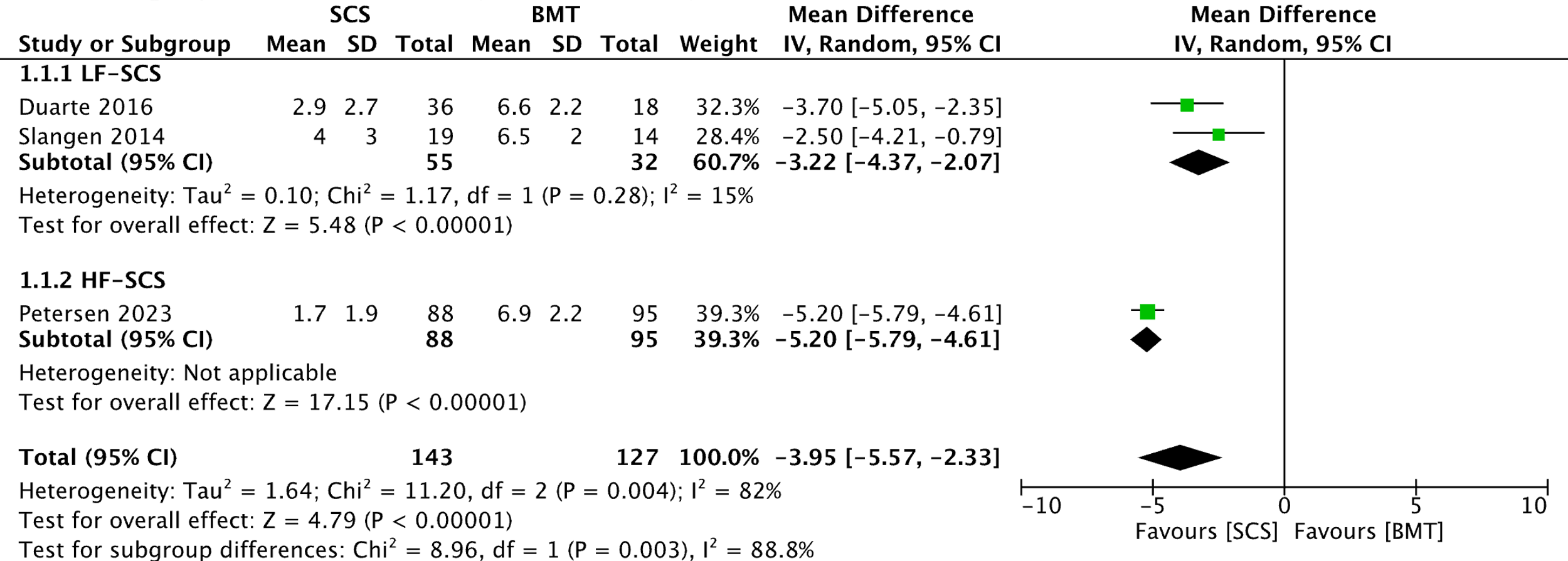


(A) Forest plot of 6-month VAS scores comparing summary effect estimates generated by random-effects models for sensitivity analysis.


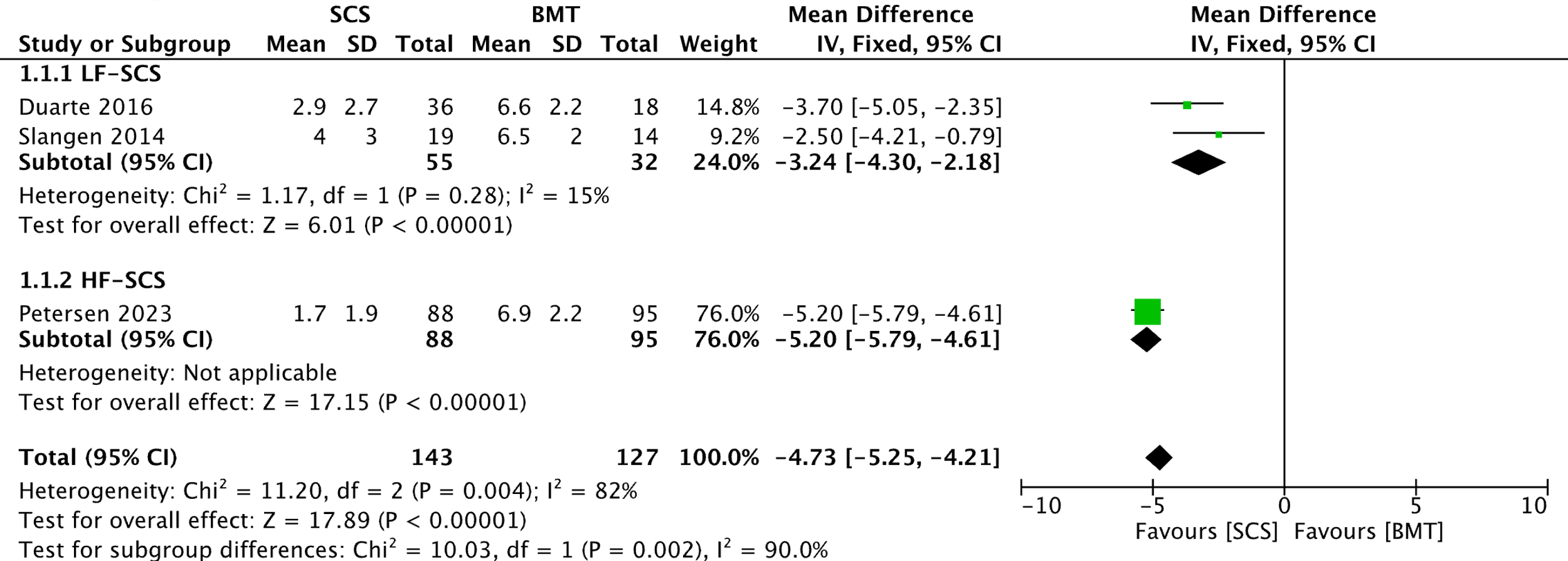


(B) Forest plot of 6-month VAS scores comparing summary effect estimates generated by fixed-effects models for sensitivity analysis.


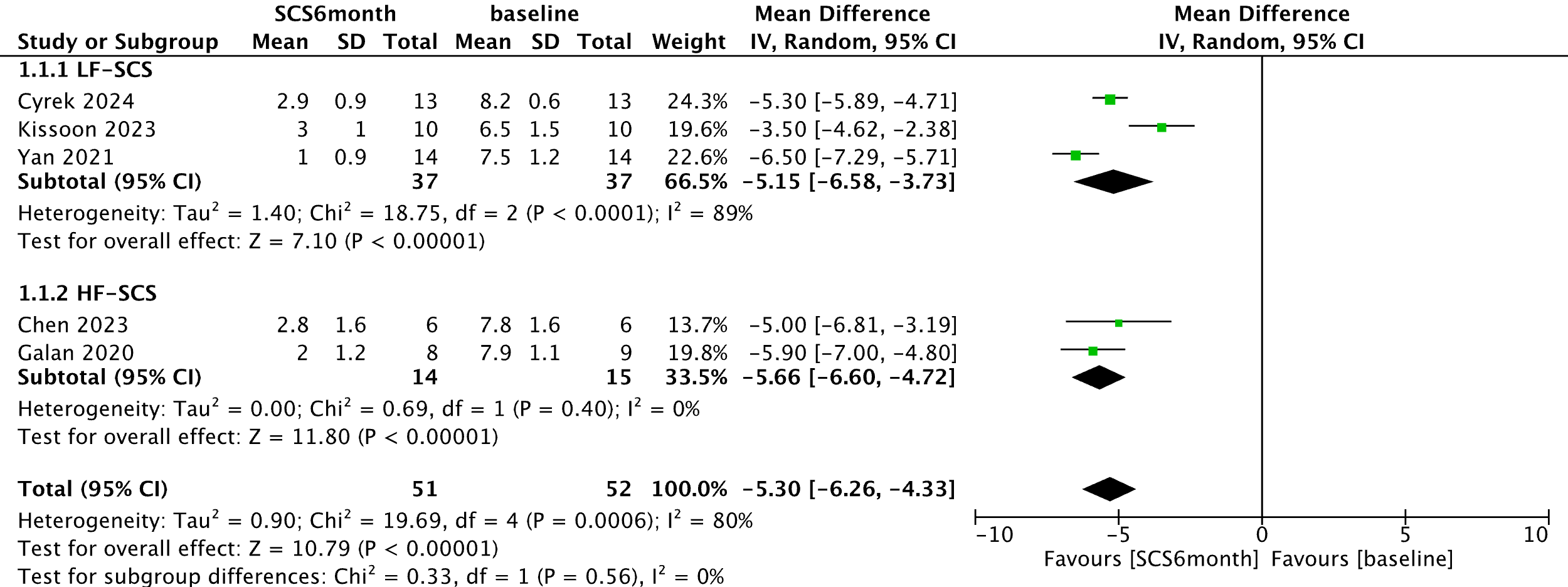


(C) Forest plot comparing pre- and post-spinal cord stimulator implantation VAS scores at 6-month follow-ups. Summary effect estimates were generated using a random-effects model with inverse-variance weighting.


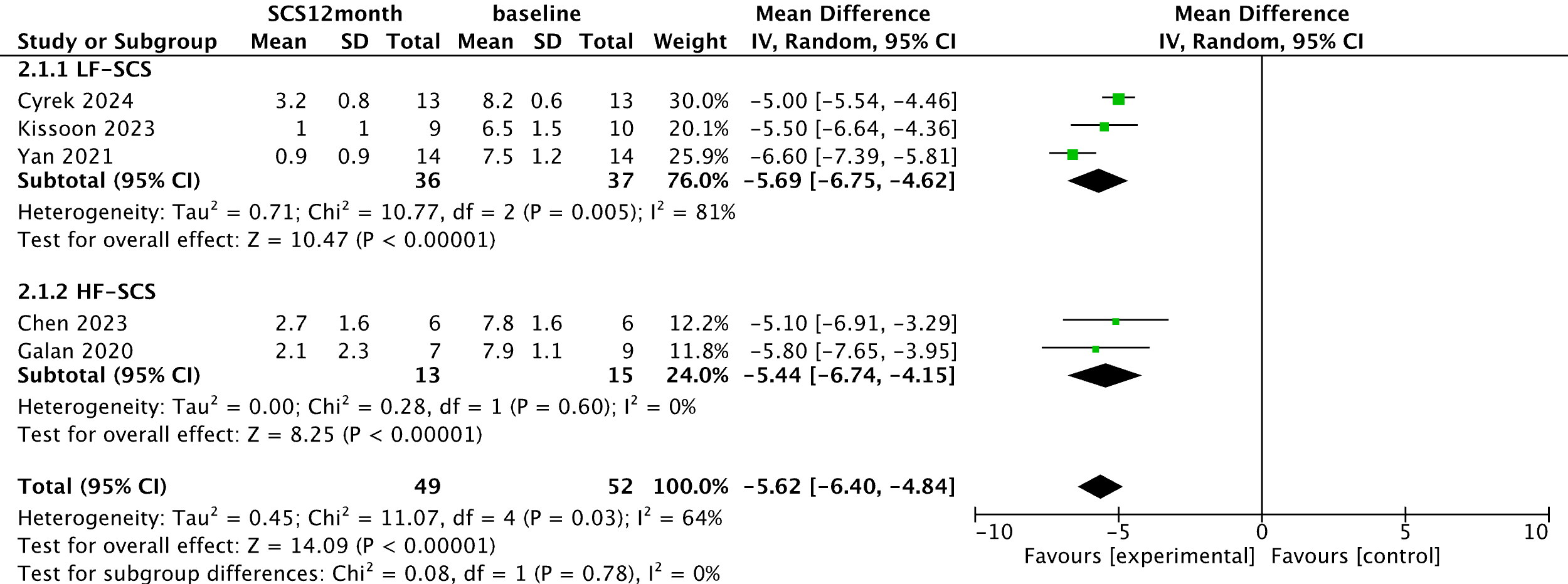


(D) Forest plot comparing pre- and post-spinal cord stimulator implantation VAS scores at 12-month follow-ups. Summary effect estimates were generated using a random-effects model with inverse-variance weighting.


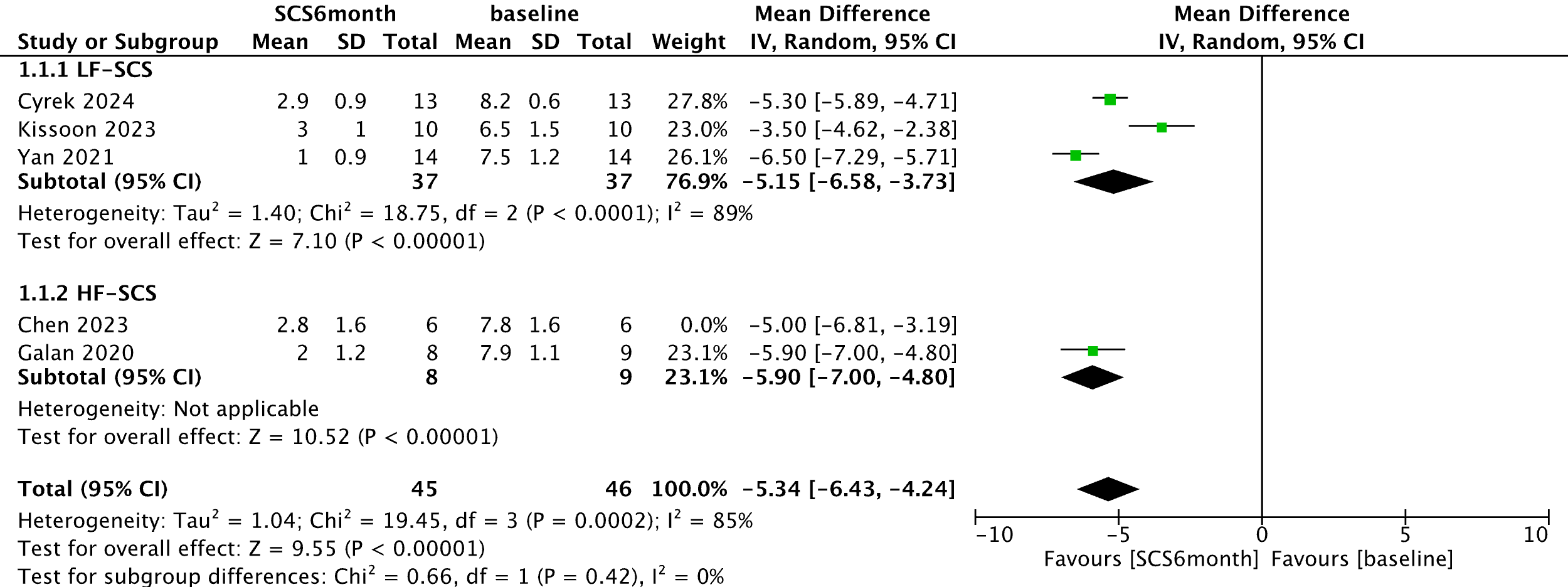


(E) Forest plot showing the sensitivity analysis excluding the study by Chen (2023). The plot compares the 6-month VAS scores of SCS to baseline.


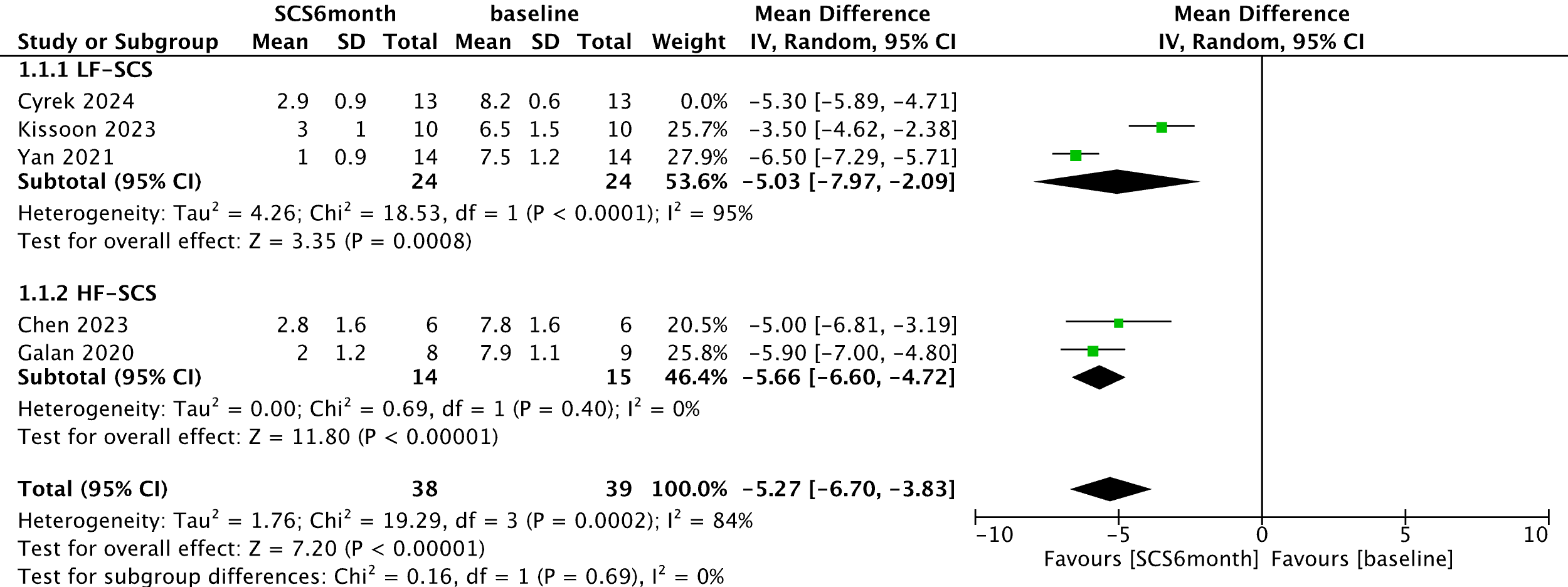


(F) Forest plot showing the sensitivity analysis excluding the study by Cyrek (2024). The plot compares the 6-month VAS scores of SCS to baseline.


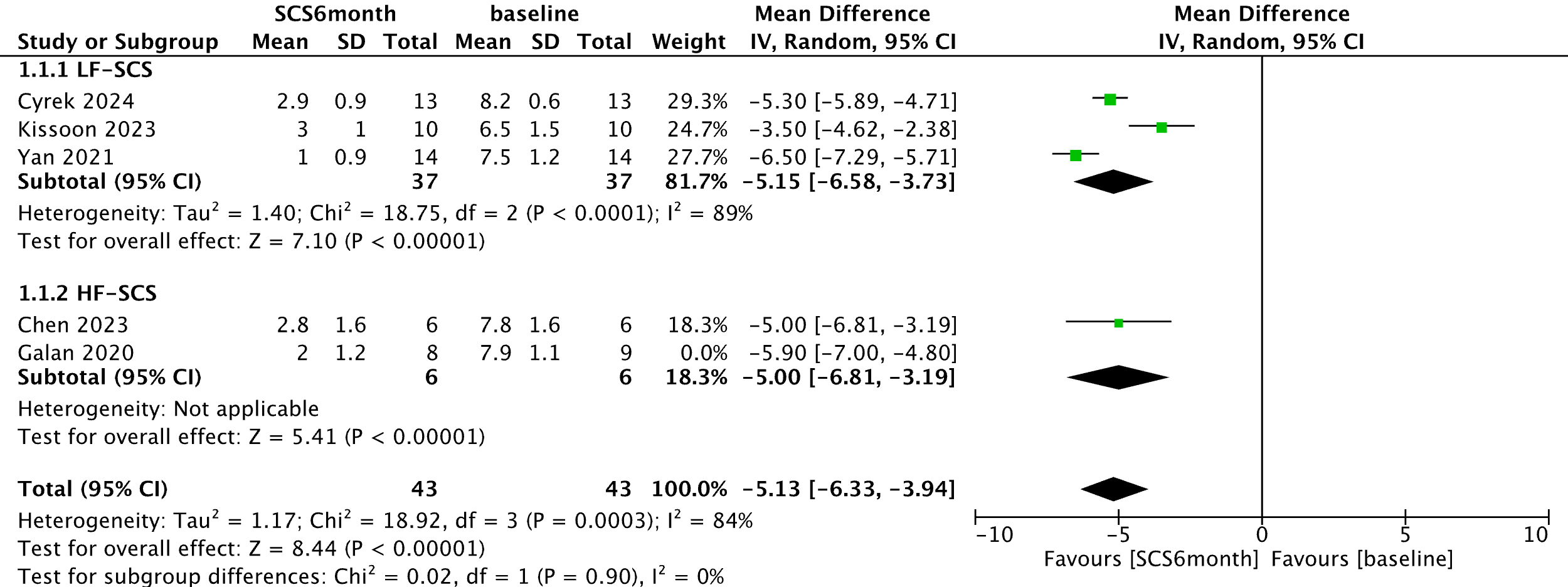


(G) Forest plot showing the sensitivity analysis excluding the study by Galan (2020). The plot compares the 6-month VAS scores of SCS to baseline.


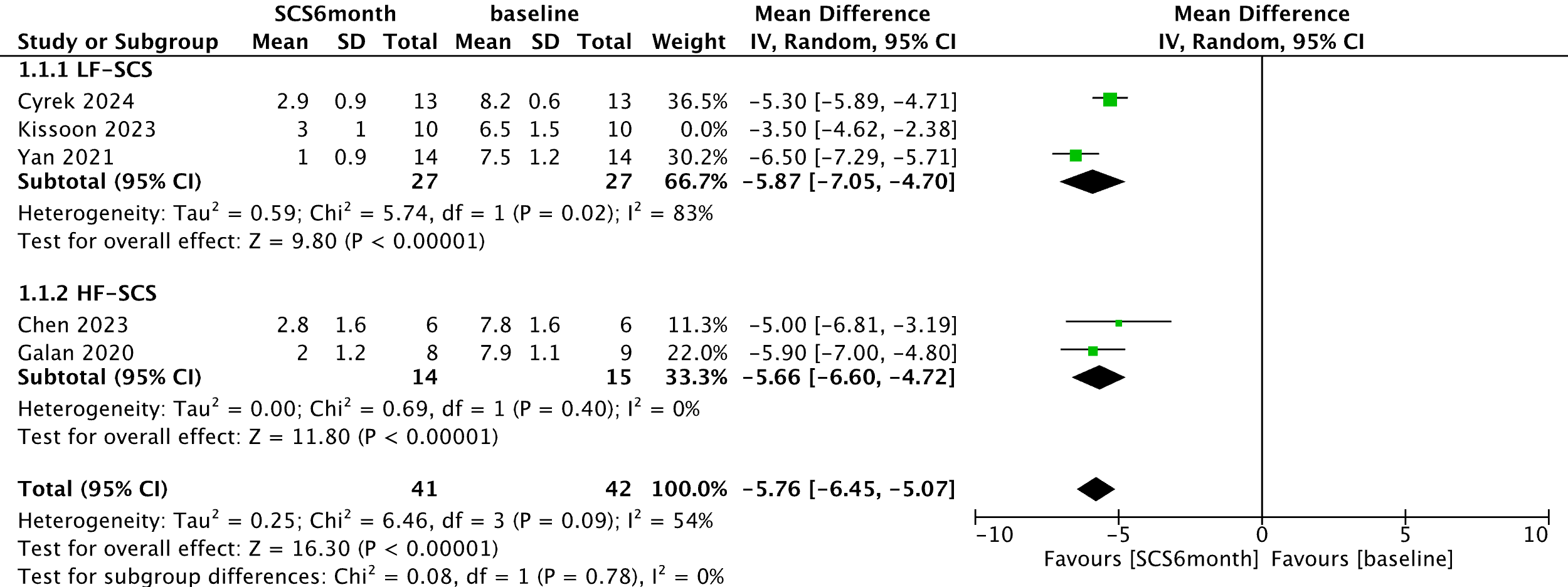


(H) Forest plot showing the sensitivity analysis excluding the study by Kissoon (2023). The plot compares the 6-month VAS scores of SCS to baseline.


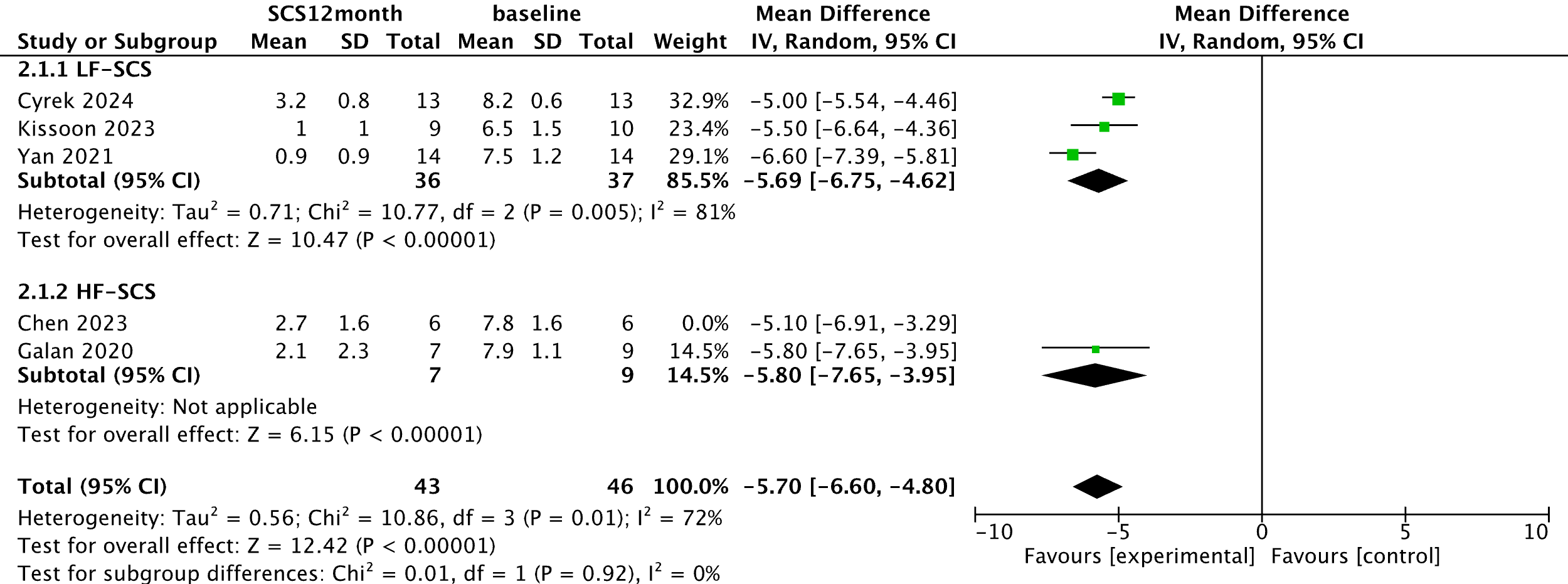


(I) Forest plot showing the sensitivity analysis excluding the study by Chen (2023). The plot compares the 12-month VAS scores of SCS to baseline.


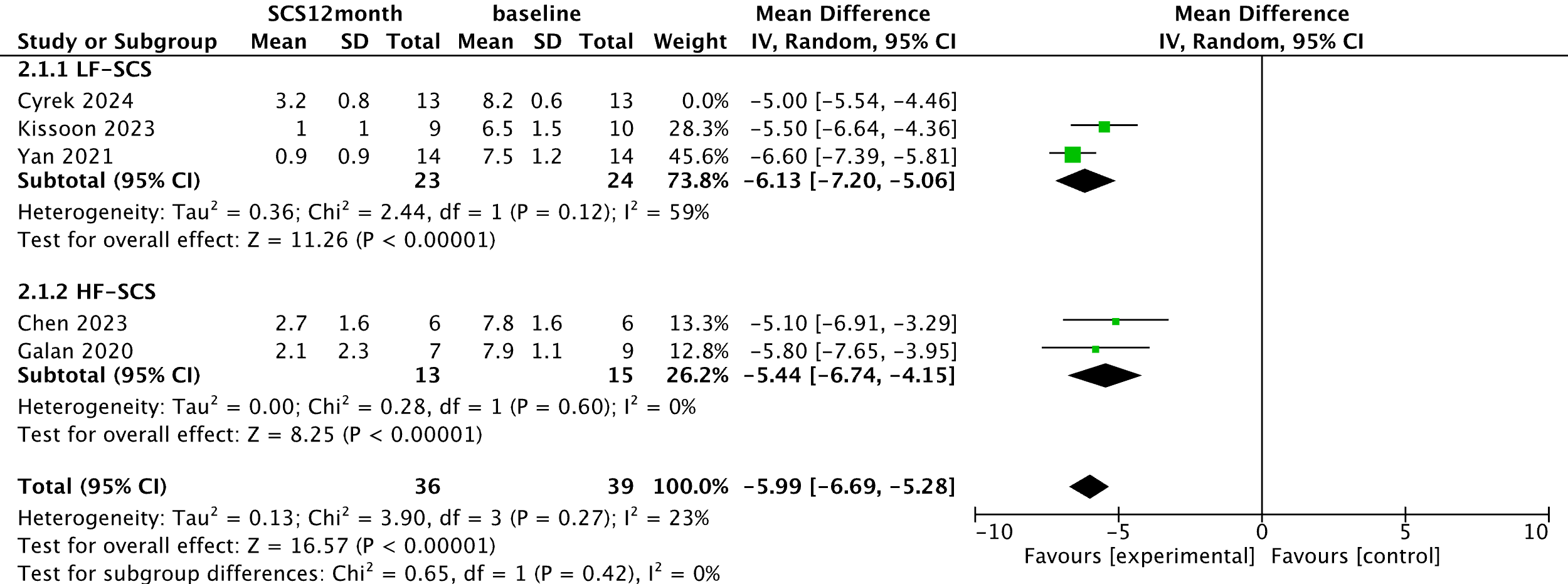


(J) Forest plot showing the sensitivity analysis excluding the study by Cyrek (2024). The plot compares the 12-month VAS scores of SCS to baseline.


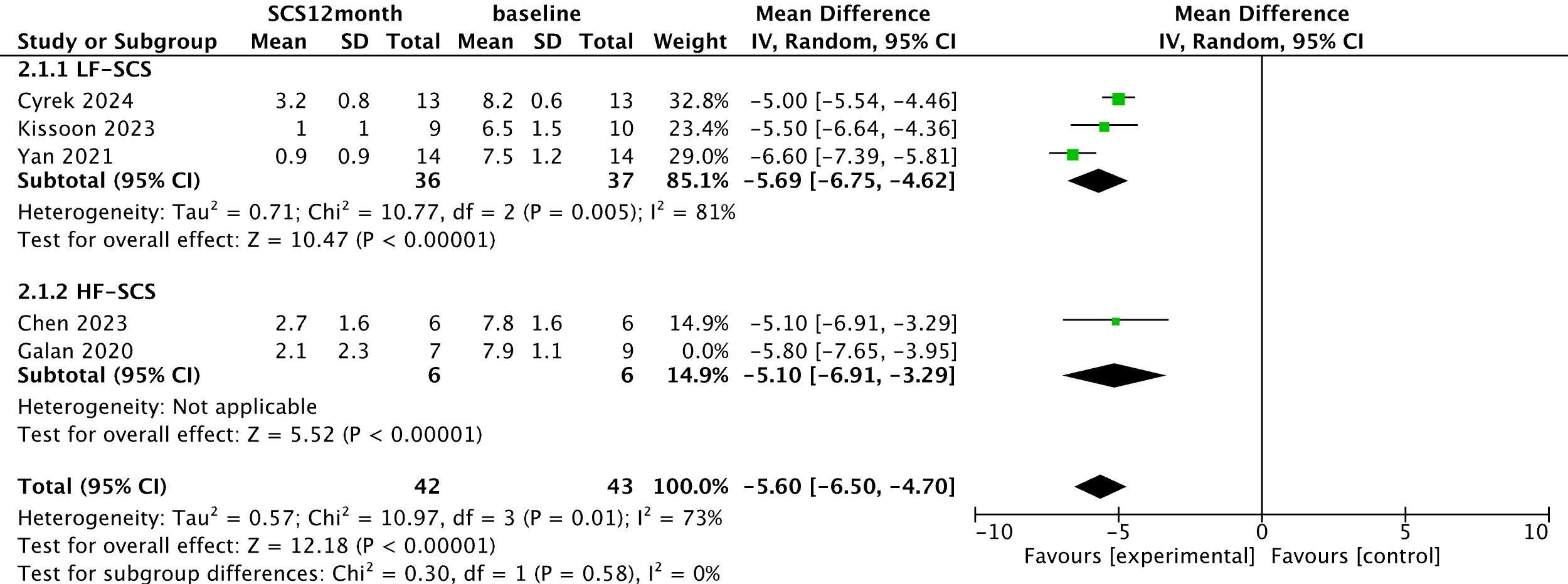


(K) Forest plot showing the sensitivity analysis excluding the study by Galan (2020). The plot compares the 12-month VAS scores of SCS to baseline.


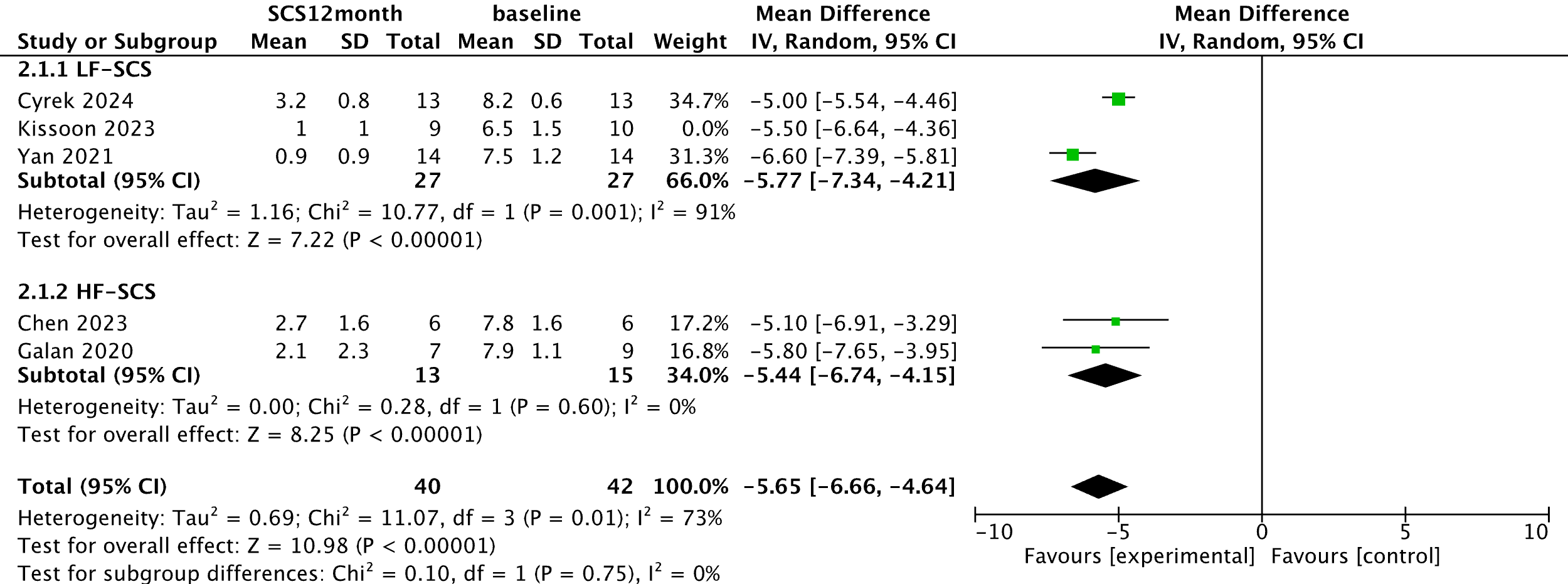


(L) Forest plot showing the sensitivity analysis excluding the study by Kissoon (2023). The plot compares the 12-month VAS scores of SCS to baseline.


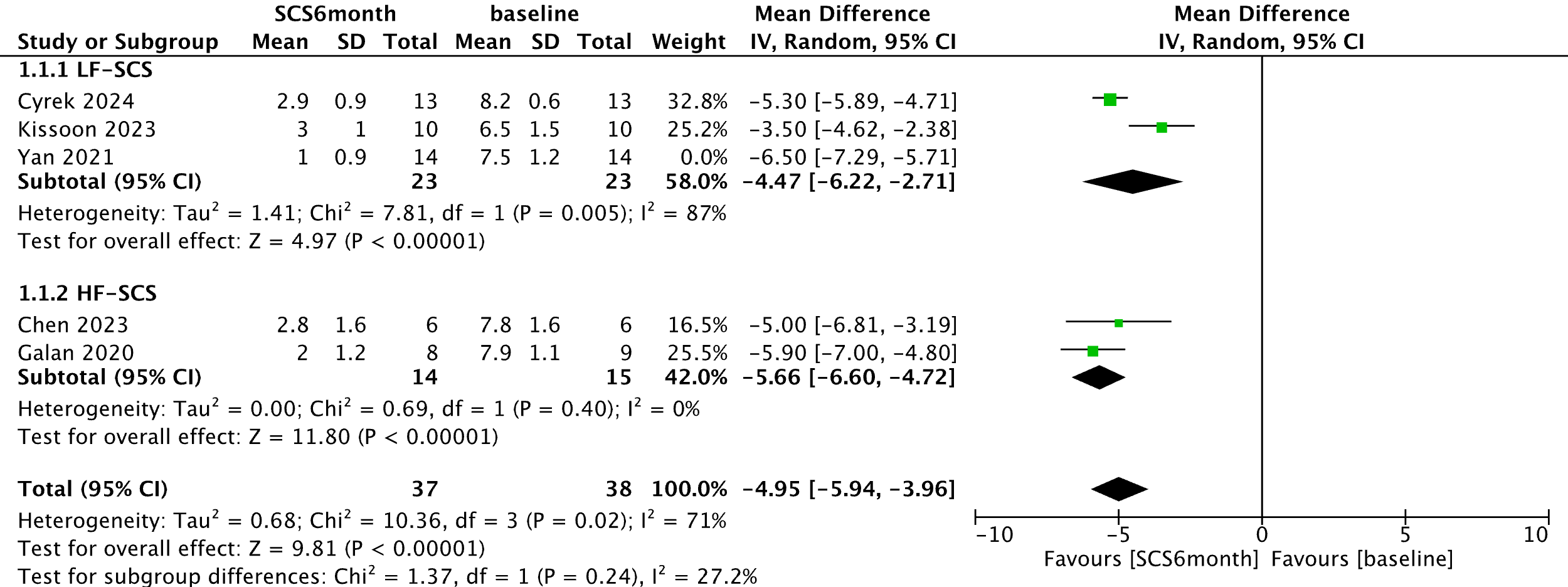


(M) Forest plot showing the sensitivity analysis excluding the study by Yan (2021). The plot compares the 6-month VAS scores of SCS to baseline.


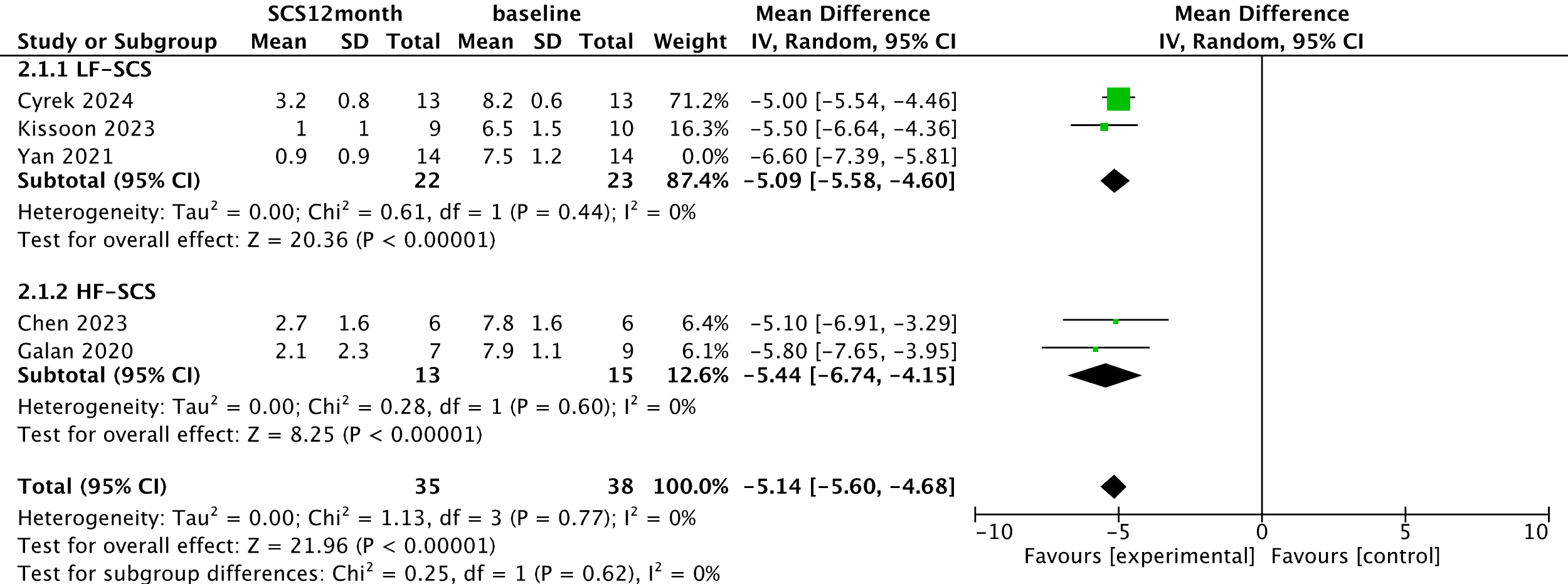


(N) Forest plot showing the sensitivity analysis excluding the study by Yan (2021). The plot compares the 1-month VAS scores of SCS to baseline.
